# Characterization of Pulmonary Vein Dimensions Using High-Definition 64-Slice Computed Tomography prior to Radiofrequency Catheter Ablation for Atrial Fibrillation

**DOI:** 10.1155/2014/179632

**Published:** 2014-06-25

**Authors:** Catherine Gebhard, Nazmi Krasniqi, Barbara E. Stähli, Bernd Klaeser, Tobias A. Fuchs, Jelena R. Ghadri, Laurent Haegeli, Thomas F. Lüscher, Philipp A. Kaufmann, Firat Duru

**Affiliations:** ^1^Department of Cardiology, Cardiovascular Center, University Hospital Zurich, Rämistrasse 100, 8091 Zurich, Switzerland; ^2^Department of Radiology, Cardiac Imaging, University Hospital Zurich, Rämistrasse 100, 8091 Zurich, Switzerland; ^3^Zurich Center for Integrative Human Physiology (ZIHP), University of Zurich, Winterthurerstrasse 190, 8057 Zurich, Switzerland

## Abstract

*Background*. Contrast-enhanced computed tomography is commonly acquired before radiofrequency catheter ablation (RFCA) for atrial fibrillation (AFib) to guide the procedure. We analyzed pulmonary vein (PV) ostial diameter and volumes on a high-definition 64-slice CT (HDCT) scanner in patients with AFib prior to RFCA. *Methods and Results*. This retrospective study included 50 patients (mean age 60.2 ± 11.4 years, 30 males) undergoing cardiac HDCT scanning before RFCA for drug refractory AFib and 50 age-, BMI-, and sex-matched controls with normal sinus rhythm undergoing HDCT. PV ostial diameter and volume were measured and calculated using a semiautomatic calliper tool. Total ostial PV volume was significantly increased in patients with AFib as compared to controls (*P* < 0.005). Similarly, total ostial PV diameter was significantly increased in AFib compared to controls (*P* < 0.001). In AFib, the largest PV volume and diameters were measured in right superior PV (*P* < 0.05 versus controls). The difference in PV volume between patients and controls was most pronounced in right superior PVs (*P* = 0.015). Right middle PVs were found more often in patients with AFib (16/50; 32%) than in normal subjects (7/50; 14%). *Conclusion*. Enlargement of PV ostial area and enlargement of volume are frequent findings in patients with drug refractory AFib. These parameters may add to the risk stratification for AFib recurrence following RFCA.

## 1. Introduction

Atrial fibrillation (AFib) is one of the most common arrhythmias with an increased prevalence with advancing age [1]. Pulmonary veins (PVs), in particular sleeves of atrial myocardium extending into the PVs, have been recognized as a major source of ectopic arrhythmogenic foci initiating AFib [2]. Pulmonary vein isolation by radiofrequency catheter ablation (RFCA) has become an effective and safe treatment option for selected patients with AFib [3, 4]. Accurate assessment of the anatomy of the PVs and the dimension of PV ostia is essential to optimize guidance of the ablation procedure [5, 6]. Cardiac computed tomography (CT) is an established imaging modality to visualize the posterior left atrium and the PV anatomy before RFCA [7, 8]. Indeed, PV ostial dimensions obtained by CT have been shown to correlate with those assessed by intracardiac echocardiography and venography [7] and introduction of high-definition CT (HDCT) may further improve such assessment.

Pulmonary vein anatomy is considered to play an important role in the initiation of AFib. Previous studies have shown that left atrial and PV remodeling including geometric alterations towards a round shape in the ostia of superior PVs occurs in patients with successful RFCA compared to those with recurrence of AFib [9]. These findings suggest that morphologic alterations of PV anatomy, in particular enlargement of PVs, may alter PV arrythmogenicity and facilitate the induction of AFib. Indeed, variations in PV anatomy are more common in patients with AFib, and diameters of PV ostia are known to be larger [10]. However, the assessment of PV diameters may be hampered by the elliptical shape of PV ostia, and assessment of PV ostial area and volume may overcome this geometric difficulty.

Hence, the aim of this study was to assess whether total PV ostial diameter and volume assessed by HDCT are larger in patients with AFib prior to RFCA compared to controls with sinus rhythm (SR), as those parameters may provide a noninvasive tool for risk stratification of AFib recurrence after RFCA.

## 2. Methods

### 2.1. Patients

This retrospective study enrolled 50 consecutive patients undergoing HDCT before RFCA for drug refractory AFib and 50 controls matched for gender, age, and body mass index with normal SR who underwent clinically indicated contrast-enhanced cardiac HDCT scanning between July 2011 and June 2012. The need to obtain written informed consent in this study was waived by the institutional review board (local ethics committee) since, according to Swiss law on clinical investigations, informed consent is not required if the nature of the study is purely observational and includes solely clinical data collection. Indications for CT in patients with SR were typical (*n* = 23) or atypical angina (*n* = 9), dyspnoea (*n* = 3), positive stress test (*n* = 8), or preoperative risk evaluation (*n* = 7). Exclusion criteria for cardiac CT examination were renal failure (glomerular filtration rate <30 mL/min), known allergy to iodine contrast material, severe claustrophobia, and pregnancy. Baseline characteristics are summarized in Table 1.

### 2.2. Cardiac CT Acquisition

All scans were performed on a 64-HDCT scanner (Discovery HD 750, GE Healthcare) with prospective electrocardiogram (ECG) triggering (75% of RR-interval), a body surface area- (BSA-) adapted contrast media bolus (Visipaque, GE Healthcare, 40–125 mL, 3.5–5 mL/s) [11–13], and i.v. *β*-blockers, if needed, to achieve a heart rate lower than 65 beats per minute and 0.4 mg sublingual nitroglycerine was administered to the control group immediately before the study. Radiation dose was calculated from the dose-length product using a conversion factor of 0.014 mSv/(mGy/cm) [14]. The scanning parameters included 64 × 0.625 mm collimation, a rotation time of 0.35 s, and body mass index- (BMI-) adjusted tube voltage (100–120 kV) and current (450–700 mA). The Discovery CT 750 HD scanner is a high-definition CT scanner equipped with a Gemstone detector. Images were acquired in high resolution mode (230 *μ*m) and reconstructed in high-definition mode (in plane resolution 0.23 × 0.23 mm, 2,496 views per rotation, and cardiac spatial resolution 18.2 lp/cm). Images were reconstructed by applying a blending factor of 30% of adaptive statistical iterative reconstruction (ASiR; GE Healthcare) according to clinical standards established in our institution. ASiR is a modified iterative reconstruction algorithm that models the photon statistics in X-ray attenuation, resulting in significant noise reduction, which improves image quality and allows reduction in radiation dose.

### 2.3. Cardiac CT Analysis

Images with a reconstruction interval of 0.6 mm were transferred to a workstation (Advantage AW 4.4 workstation, GE Healthcare) with 3D capabilities. Since it can be challenging to identify the ostium in 3D reconstructions, the PV ostial dimensions were first evaluated using a 2-dimensional viewing mode assisted by oblique and curved multiplanar reconstructions (Figure 4). When PV ostia had been viewed and clearly identified, measurement of minimal and maximal PV diameter and area at the level of the ostium was obtained by using an automatic tool. Then, 3D volume-rendered images were used for depiction of pulmonary vein anatomical variants and branching patterns. Normal anatomy was defined as presence of two septal or right and two lateral or left separate PV ostia. A left common ostium (LCO) was defined when the superior and inferior left PV joined >5 mm before entering the left atrium. A right common ostium (RCO) was defined when the superior and inferior right PV joined >5 mm before entering the left atrium. A right middle pulmonary vein (RMPV) was defined as accessory, separate ostium for the middle-lobe vein. Other anatomical variants were not observed in our population. The volume was then measured and automatically calculated in 3D reconstruction from the previously determined point of inflection between the PV and atrial wall along the course of the PV to the midpoint of the PV 1 cm distally of the ostium. To standardize the analysis, images were displayed with a fixed window level at 100 HU and a window width at 800 HU. Similarly, the LA area was traced by using 3D volume-rendered images and semiautomated circumference measurement by excluding PVs at their ostia and the LA appendage at its base. LA volume was calculated by using an automatic tool. Volumes were reported independently and also indexed to body surface area.

### 2.4. Statistical Analysis

Quantitative variables were expressed as mean ± SD or mean ± SEM and categorical variables were expressed as frequencies or percentages. Normality was tested with the Kolmogorov-Smirnov test. Comparisons between the two groups were performed using Student's *t*-test for continuous variables with normal distributions and the Mann-Whitney *U* test for continuous variables with nonnormal distributions. All analyses were performed with statistics software (SPSS version 20.0 for Microsoft Windows). A two-tailed *P* value of <0.05 was deemed significant.

## 3. Results

### 3.1. Patient Characteristics

Cardiac HDCT scans from 50 consecutive patients (mean age 60.1 ± 11.4 years, mean BMI 27.3 ± 4.3 kg/m^2^, 30 males) undergoing RFCA for drug refractory AFib and from 50 controls with normal sinus were analyzed. Controls were adequately matched for gender, age, and body mass index (*P* = NS). A total contrast amount of 102.3 ± 1.8 mL (AFib) and 89.3 ± 2.4 mL (controls) was administered (Table 1; *P* = NS). Eight (16%) patients in the AFib group were with AFib during image acquisition. The average effective radiation dose was 2.1 ± 0.3 mSv (AFib) and 2.0 ± 0.4 mSv (controls, *P* = NS), respectively (Table 1). Baseline characteristics are summarized in Table 1.

### 3.2. Total Pulmonary Vein Ostial Diameter and Volume Are Increased in Patients with AFib

**Figure 1:**
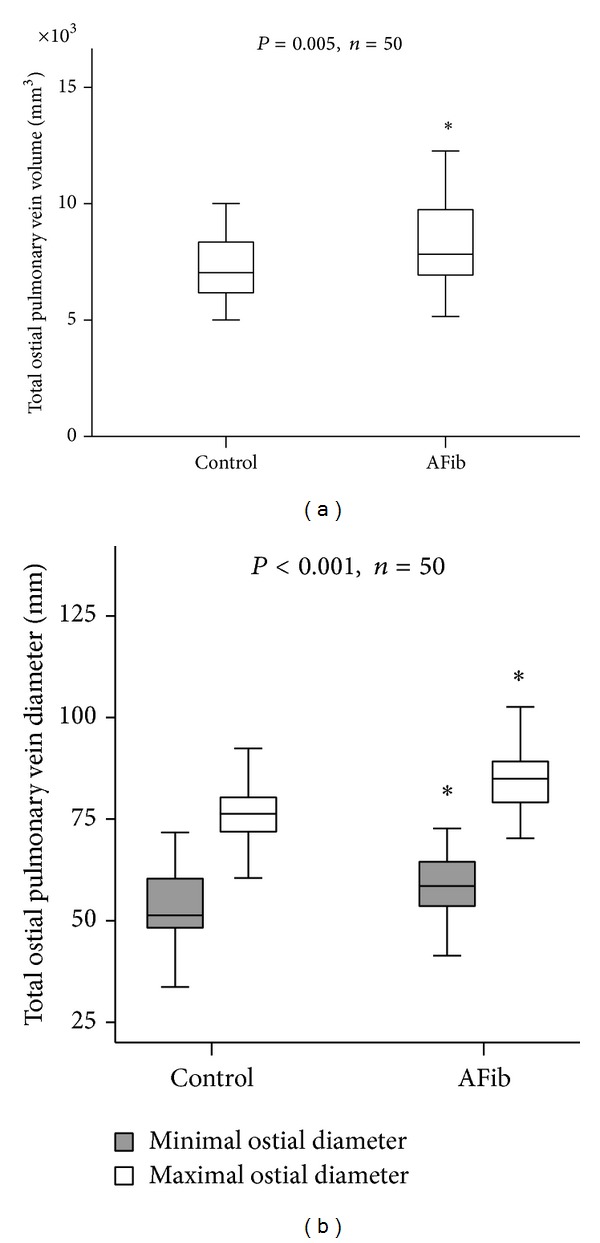
(a) Boxplots of total pulmonary vein (PV) volume (mm^3^) in patients with atrial fibrillation (AFib) compared to controls with sinus rhythm (SR). (b) Total ostial pulmonary vein (PV) diameter (mm) in patients with atrial fibrillation (AFib) compared to healthy controls with sinus rhythm (SR). Data are presented as mean ± SD; ^∗^ *P* < 0.05.

Total PV ostial volume was significantly increased in patients with AFib as compared to controls: total PV ostial volume was 8356.7 ± 296.5 mm^3^ in patients with AFib compared to 7309.02 ± 213.232 mm^3^ in controls (*P* < 0.005, Figure 1(a)). Similarly, total minimal and maximal ostial PV diameters were significantly increased in AFib compared to controls (59.2 ± 1.4 mm and 85.2 ± 1.3 mm versus 53.3 ± 1.2 mm and 76.9 ± 1.1 mm, resp.; *P* < 0.001, Figure 1(b)).

### 3.3. The Largest Differences in Volumes between AFib and Controls Are Found in Superior PVs

**Figure 2:**
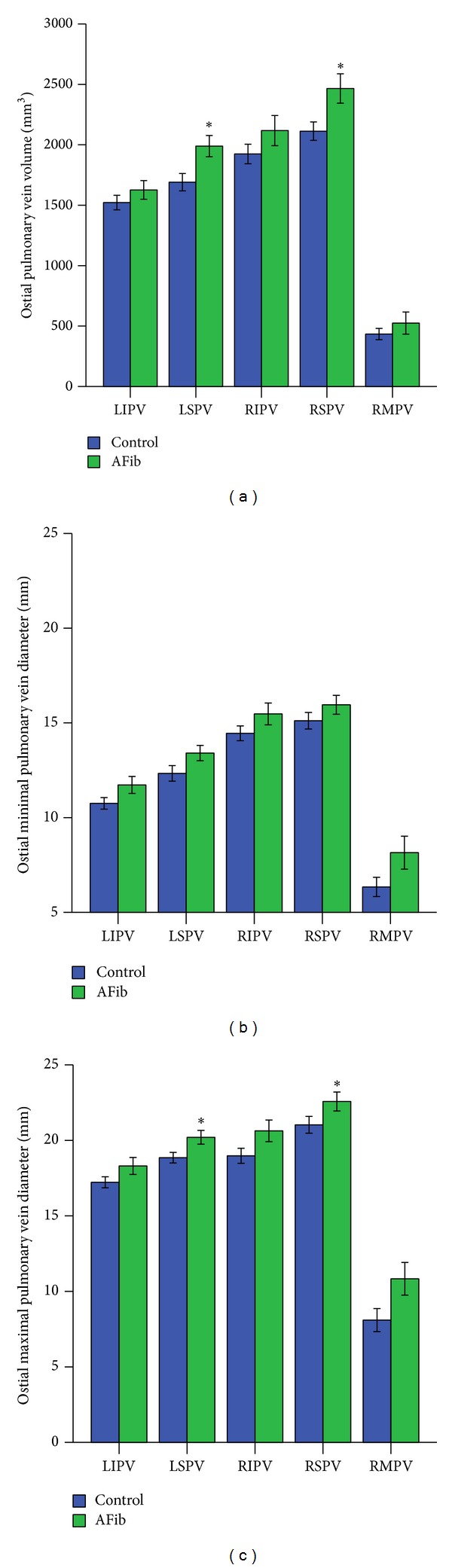
(a) Distribution of pulmonary vein (PV) volume (mm^3^) in patients with atrial fibrillation (AFib) compared to controls with sinus rhythm (SR). (b) Distribution of minimal pulmonary vein (PV) diameter (mm) in patients with atrial fibrillation (AFib) compared to healthy controls with sinus rhythm (SR). (c) Distribution of maximal pulmonary vein (PV) diameter (mm) in patients with atrial fibrillation (AFib) compared to healthy controls with sinus rhythm (SR). LIPV, left inferior pulmonary vein; LSPV, left superior pulmonary vein; RIPV, right inferior pulmonary vein; RSPV, right superior pulmonary vein; RMPV, right middle pulmonary vein. Data are presented as mean ± SEM; ^∗^ *P* < 0.05.

In patients with AFib, the largest PV ostial volume and maximal diameter were measured in right superior PV (2465.9 ± 860.4 mm^3^ and 22.6 ± 0.6 mm, resp., versus 2112.7 ± 544.5 mm^3^ and 21.0 ± 0.6 mm in controls, Figures 2(a) and 2(c); *P* < 0.05). In left superior PV, volume and diameter were 1989.02 ± 621.5 mm^3^ and 20.2 ± 0.5 mm in AFib versus 1720.5 ± 515.6 mm^3^ and 18.9 ± 0.4 mm in controls (*P* < 0.05, Figures 2(a) and 2(c)). Accordingly, the difference in PV volume between AFib patients and controls was most pronounced in right superior PV (Δvolume = 14.4%; *P* = 0.015, Figure 2(a)) and left superior PV (Δvolume = 14.5%; *P* < 0.01). In right inferior PV, volume was 2117.9 ± 883.9 mm^3^ in AFib versus 1924.4 ± 578.2 mm^3^ in controls (Δvolume = 9.2%; *P* = NS, Figure 2(a)). In right middle PV, volume was 525.0 ± 354.8 mm^3^ in AFib versus 433.6 ± 123.4 mm^3^ in controls (Δvolume = 17.5%; *n* = 22; *P* = NS, Figure 2(a)). In left inferior PV, volume was 1626.4 ± 544.5 mm^3^ in AFib versus 1522.0 ± 433.7 mm^3^ in controls (Δvolume = 7%; *P* = NS, Figure 2(a)). Ostial volume and maximal diameter of right middle PVs were slightly increased in AFib as compared to controls: right middle PV volume was 525.0 ± 354.8 mm^3^ in AFib versus 433.6 ± 123.4 mm^3^ in controls, while ostial right middle PV (maximal) diameter was 8.2 ± 0.8 mm in AFib versus 6.3 ± 0.5 mm^2^ in controls. However, this difference was not significant. No difference was observed in minimal PV ostial diameters between groups (*P* = NS, Figure 2(b)).

**Figure 3:**
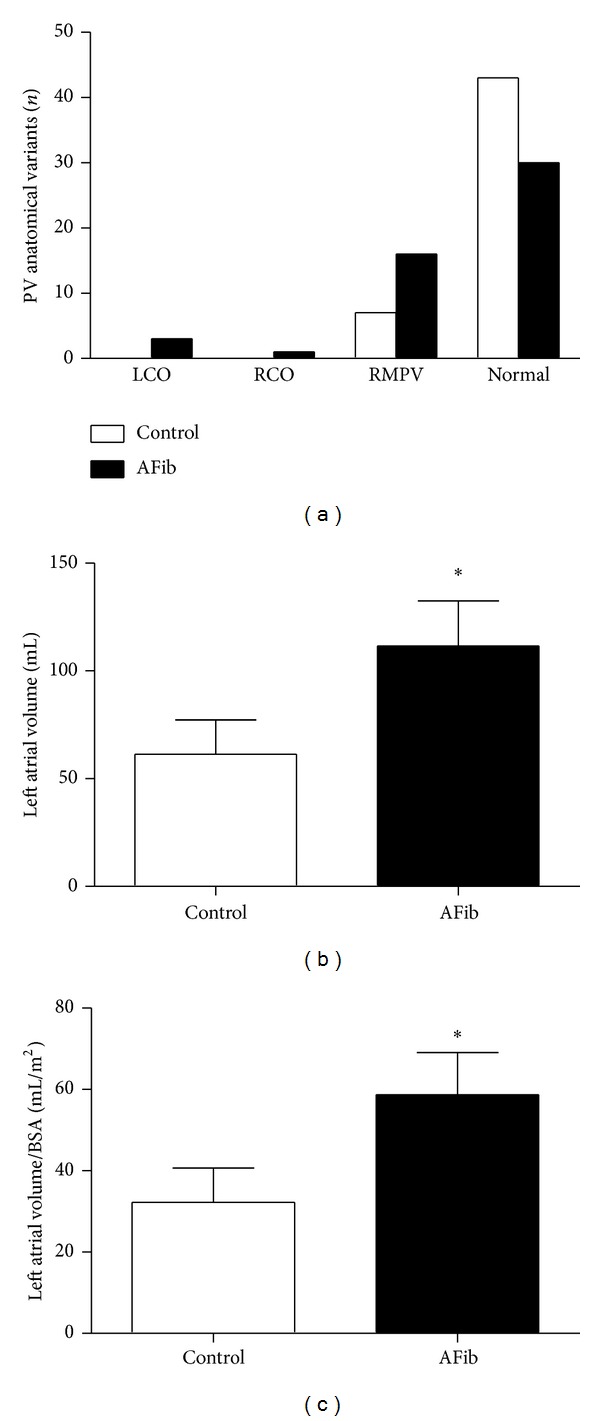
(a) Number of patients with a right middle pulmonary vein (RMPV), common left ostium (LCO), and common right ostium (RCO). Patients with AFib (atrial fibrillation) versus controls with SR (sinus rhythm). *N* = number of patients; total number of patients in each group = 50. (b) Volume of left atrium after exclusion of the pulmonary veins and left atrial appendage. (b) Left atrial volume in controls and patients with AFib. (c) Left atrial volume indexed to body surface area (BSA) in controls and patients with AFib; ^∗^ *P* < 0.05.

**Figure 4:**
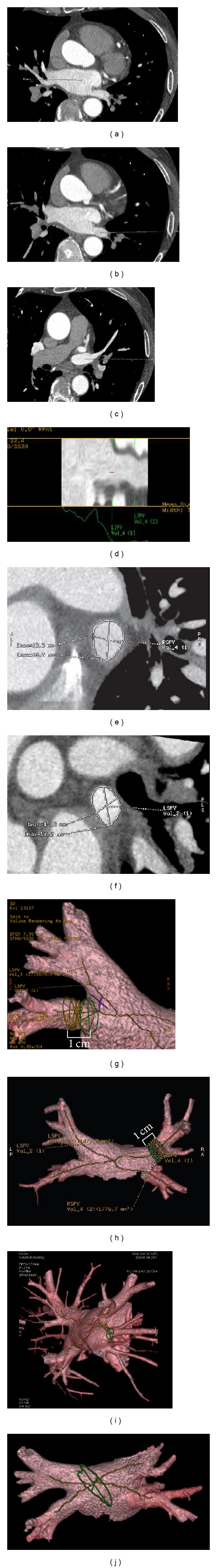
(a–c) Identification and labelling of left atrium and pulmonary vein ostia in 2-dimensional (axial) viewing. (d) Confirmation of point of inflection between pulmonary vein and left atrial wall in curved multiplanar reconstructions. (e-f) Following clear identification of ostia, measurement of minimal and maximal ostial pulmonary vein diameter in oblique view. (g-h) Three-dimensional volume-rendered reconstruction: the previously identified ostium was used as a starting point for volume measurements along the course of the PV to the midpoint of the PV 1 cm distal of the ostium. (i) Depiction of pulmonary vein anatomy and branching pattern by using 3D volume-rendered images. (j) Tracing and labelling of left atrium for left atrium volume measurements by using 3D rendered images. LIPV, left inferior pulmonary vein; LSPV, left superior pulmonary vein; RIPV, right inferior pulmonary vein; RSPV, right superior pulmonary vein; RMPV, right middle pulmonary vein.

Conventional PV anatomy, with 4 separate PVs, was present in 73% of patients (Figure 3). Anatomical variants were observed more often in patients with AFib (42%) compared to controls (14%, *P* < 0.05). The most common observed PV variation was a right-sided middle accessory PV in 16 patients (32%) with AFib and 7 controls (14%, *P* < 0.05); the second most frequent PV variation was a common left ostium (LCO) which was present in 4 patients with AFib (8%). No patients had a left accessory PV, and only one patient with AFib (2%) had a common right ostium (RCO).

### 3.4. Left Atrial Volume Is Increased in Patients with AFib

Left atrial (LA) volume was assessed from prospectively ECG-gated (75% of RR-interval), contrast-enhanced cardiac volumes by using 3D volume-rendered images with semiautomated circumference measurement. The mean LA volume in the total study population was 85.7 ± 18 mL with a range of 37–172 mL. LA volume was significantly smaller in control patients (62.3 ± 161 mL) than that in patients with AFib (111.6 ± 21.3 mL, *P* = 0.01). Similarly, LA volume indexed to body surface area (BSA) was smaller in control patients (32.26 ± 8.4 mL/m^2^) than in patients with AFib (58.7 ± 10.3 mL/m^2^,  *P* = 0.03).

## 4. Discussion

In this study, we demonstrate that total PV ostial volume and diameter are significantly increased in patients with drug refractory AFib compared to controls. This increase in PV ostial volume and diameter is most pronounced in superior PVs with the largest difference measured in right superior PVs. Further, we confirm that PV anatomy is different in patients with AFib with a higher frequency of right middle PVs and left common ostium.

Usually, four PVs drain into the left atrium. The superior PVs enter the left atrium from a superior, anterior, and lateral direction and the inferior ones from the posterior direction [15]. It is well known that there is considerable variation in PV anatomy and anatomical alterations of PVs, including common ostia, and additional veins have been detected in 18–45% of patients with AFib. However, the reported frequency of such anatomical variations has wide dispersion, which can in part be explained by variability in definitions of normality, and methodological differences. We observed an increased incidence of RMPV and LCO in patients with AFib. This distribution of anatomical variants in our study is very consistent with prior studies that have reported an additional RMPV or a LCO more frequently in patients with AFib compared to controls [16–18]. However, controversies exist regarding whether right-sided anatomical variants are associated with a higher risk of AFib recurrence following radiofrequency catheter ablation. While some studies did not find a relation between PV anatomy and AFib recurrence, others reported an increased incidence of AFib recurrence in normal PV anatomy compared to variant anatomy, or a higher AFib recurrence rate in anatomical variants [6, 17, 19–24]. Thus, larger clinical studies will be required to establish whether anatomical variants are a risk factor for AFib recurrence.

There is clear evidence that enlarged LA dimensions are more frequently observed in patients with AFib and that larger LA dimensions are associated with a higher risk of AFib recurrence following ablation [6, 25]. However, while LA dimensions have been well described in patients with AFib, less information is available if such morphological changes also occur in the PVs. To the best of our knowledge, to date, no study has measured ostial PV volumes in patients with AFib and only few studies have directly compared PV characteristics in patients with and without AFib. Consistent with our results, these authors report larger PV ostial diameters in patients with AFib compared to controls [10, 16, 20, 26]. Further, superior PVs seem to have a larger ostial diameter than inferior PVs, a phenomenon that was also observed in the present study [27]. Remarkably, enlargement of superior PVs has been described to be an independent risk factor for AFib recurrence following radiofrequency catheter ablation [28].

Previous investigators have measured PV diameters by using CT only in one specified plane, usually at the ostium in anterior-posterior and inferior-superior directions [10]. It is often difficult to define the border between left atrium and pulmonary veins by 2D analysis; thus, we used 3D reconstructed models and performed volume measurements in all PVs starting from the point of inflection between the PV and atrial wall along the course of the PV to the midpoint of the PV 1 cm distally of the ostium. In addition, we used oblique and curved multiplanar reconstructions to confirm the borders of both left atrium and PVs. Since PV ostia are considered to have an elliptical shape with the long axis in vertical orientation [29], 3D measurement and calculation of PV areas and volumes may cope more precisely with the elliptical shape of the ostia and may be a more suitable diagnostic tool to detect anatomical differences in AFib compared with diameter measurements and may improve diagnostic accuracy.

Limitations of this study are its retrospective design limited to a single center experience. Further, the study population size is limited, which is in part counterbalanced by optimal matching of the control group. In addition, the prognostic value of our findings remains to be elucidated, and further studies are needed to assess whether the observed alterations in PV anatomy contribute to the complex pathophysiology of AFib or whether larger PV ostia are a cause, a consequence, or just an epiphenomenon of atrial structural remodelling in AFib.

In conclusion, we found that PV ostial diameter and PV volume were significantly larger in patients with drug refractory AFib than in controls and that this difference was most pronounced in right superior PVs. This finding suggests that an assessment of PV ostial volume may add to the risk stratification for AFib recurrence following RFCA and may be incorporated into the preprocedural evaluation of patients being considered for AFib ablation.

## Figures and Tables

**Table 1:** Patient characteristics (patient baseline and CT acquisition characteristics).

	AFib *n* = 50	SR *n* = 50
Male sex, number of patients (of total)	30 (50)	30 (50)
Age (years)	60 ± 11	59 ± 10
BMI (kg/m^2^)	27.9 ± 4.6	27.2 ± 4.3
mSv	2.1 ± 0.3	2.0 ± 0.4
Heart rate (bpm)	55.5 ± 2.1	69.2 ± 1.2
Tube voltage (kV)	113 ± 1.2	115 ± 1.3
Tube current (mA)	645.7 ± 4.8	632.9 ± 5.0
Contrast volume (mL)	102.3 ± 1.8	89.3 ± 2.4
Beta-blocker (mg)	7.5 ± 1.4	0

Values are given as mean ± standard deviation (SD). BMI: body mass index; SR: sinus rhythm; AFib: atrial fibrillation.
